# Maltreatment and Mental Health Outcomes among Ultra-Poor Children in Burkina Faso: A Latent Class Analysis

**DOI:** 10.1371/journal.pone.0164790

**Published:** 2016-10-20

**Authors:** Leyla Ismayilova, Eleni Gaveras, Austin Blum, Alexice Tô-Camier, Rachel Nanema

**Affiliations:** 1 The University of Chicago, Chicago, United States of America; 2 Trickle Up, Ouagadougou, Burkina Faso; University College London, UNITED KINGDOM

## Abstract

**Objectives:**

Research about the mental health of children in Francophone West Africa is scarce. This paper examines the relationships between adverse childhood experiences, including exposure to violence and exploitation, and mental health outcomes among children living in ultra-poverty in rural Burkina Faso.

**Methods:**

This paper utilizes baseline data collected from 360 children ages 10–15 and 360 of their mothers recruited from twelve impoverished villages in the Nord Region of Burkina, located near the Sahel Desert and affected by extreme food insecurity. We used a Latent Class Analysis to identify underlying patterns of maltreatment. Further, the relationships between latent classes and mental health outcomes were tested using mixed effected regression models adjusted for clustering within villages.

**Results:**

About 15% of the children in the study scored above the clinical cut-off for depression, 17.8% for posttraumatic stress disorder (PTSD), and 6.4% for low self-esteem. The study identified five distinct sub-groups (or classes) of children based on their exposure to adverse childhood experiences. Children with the highest exposure to violence at home, at work and in the community (Abused and Exploited class) and children not attending school and working for other households, often away from their families (External Laborer class), demonstrated highest symptoms of depression and trauma. Despite living in adverse conditions and working to assist families, the study also identified a class of children who were not exposed to any violence at home or at work (Healthy and Non-abused class). Children in this class demonstrated significantly higher self-esteem (b = 0.92, SE = 0.45, p<0.05) and lower symptoms of trauma (b = -3.90, SE = 1.52, p<0.05).

**Conclusions:**

This study offers insight into the psychological well-being of children in the context of ultra-poverty in Burkina Faso and associated context-specific adverse childhood experiences. Identifying specific sub-groups of children with increased exposure to life stressors has implications for program developers. Study findings indicate a further need to explore the mental health consequences of traumatic experiences within the context of ultra-poverty and to develop integrated economic and psychosocial interventions that prevent or mitigate childhood adversities linked with the family-level poverty and violence in the family.

## Introduction

Children who experience adversities during early development are likely to have poorer mental health later in life than those who were raised in supportive environments [1–3]. Early life stressors such as abuse, extreme poverty, family violence, and related adverse experiences affect children’s brain and behavioral functioning and may disrupt the child’s stress regulatory system [3,4]. Although the link between Adverse Childhood Experiences (ACEs) and child psychopathology is well documented, most studies focused on types of adversities experienced by children in developed countries. Most of the world’s youth, however, reside in developing nations and limited data are available for additional types of adversity faced by these children [5]. The UN’s Sustainable Development Goals (SDGs) included mental health (Target 3.4) in the 2030 agenda and elevated the importance of preventing and promoting mental well-being to a global priority [6]. To achieve this goal and guide the development of mental health policies and interventions, it is crucial to understand the life stressors undermining children’s emotional well-being. Few studies, however, have investigated the childhood stressors and risk factors contributing to child mental health outcomes in Sub-Saharan Africa; very few have investigated them in Francophone West African countries such as Burkina Faso [2,7–9].

### Child Mental Health in Sub-Saharan Africa

Adults in Sub-Saharan Africa show an elevated prevalence of depression and posttraumatic stress disorder (PTSD) compared to Western countries [8]. Research on the etiology of these disorders, which often originate in childhood, and on the contribution of adverse early childhood experiences is limited [8,10]. A systematic review from six African countries (Ethiopia, Kenya, Nigeria, Uganda, Democratic Republic of Congo/DRC, and South Africa) reports a wide range of prevalence rates (2.7–27.0%) for child psychopathology [8]. Significant heterogeneity in the prevalence estimates of common mental health disorders is often attributed to methodological factors (sample representativeness, sample frame, the use of diagnostic interview and definition of functional impairment) and it is unclear whether there are systematic differences in overall prevalence rates among different geographic areas [11]. Nevertheless, most studies in the Sub-Saharan region of Africa were conducted primarily in predominantly English-speaking or East African countries and may not be generalizable to Francophone West African countries with different historic, social and cultural characteristics [8]. Mostly, West Africa underwent French colonization and has had a more significant Islamic influence due to its greater proximity to the Arab North. Furthermore, available studies in Sub-Saharan Africa have investigated risk factors for mental health problems mainly among at-risk children, who are orphans [12–16], HIV positive or have family members with HIV [10,17–21], or who are exposed to or engaged in war or conflict [22–24].

Relatively few studies have examined the relationship between childhood adversities and mental health outcomes among children living with their parents in the context of ongoing ultra-poverty and not under crisis circumstances such as HIV epidemic or conflict [12,25].

### Violence Exposure and Child Mental Health

Literature from high-income countries has documented well that exposure to physical violence in childhood has lifelong health consequences [3,26–30]. In the case of family physical violence, however, some researchers have questioned to what extent exposure to corporal punishment affects child mental health across different cultural contexts [26,31], particularly where such punishment is highly prevalent and normative. Some studies have found that the negative impact of corporal punishment is mitigated in communities where corporal punishment is common [32,33].

Studies conducted in the Sub-Saharan African countries of Kenya, Nigeria, South Africa and Tanzania have found that many children are exposed to high levels of violence, including family violence [34–42]. In Nigeria, 44.8% of children reported been hit repeatedly with an implement, often resulting in injures such as bruises and black eyes, and 16.8% reported experiencing emotional abuse within the home [35]. In Tanzania, parents often employ harsh physical and emotional discipline practices and believe they do no harm to their children [43]. In collective societies, parents view their approach to child rearing as authoritarian and adult-centered as opposed to more ‘permissive’ parenting style in Western cultures that puts a child’s needs at the center [44,45]. For cultural and economic reasons, African parents teach their children that the needs of the family are above children’s individual desires, and children are expected to follow responsibilities set by adults such as helping with a family business a farm, or taking care of the household and younger or elder family members. Fearful that their children may grow up lazy and disrespectful, parents believe that strict parenting practices will teach personal struggle, moral discipline, and perseverance in the face of harsh adversity [45].

### Other Childhood Adversities in the Context of Burkina Faso

Some of the most significant social determinant of child psychopathology also include socioeconomic deprivation [46,47] as well as other forms of childhood adversity associated with low socioeconomic status such as child labor and early marriage [8,48].

#### Poverty and food insecurity

Burkina Faso is one of 48 Least Developed Countries (LDC) in the world, with 17.6 million population [49]. The Nord Region of Burkina Faso, where the study was conducted, is located in the Sahel Desert and is characterized by even higher levels of ultra-poverty and extreme food insecurity, especially during the dry season which typically lasts from October through May [50,51]. In low- and middle-income countries, poverty is associated with poor mental health, but the association is linked only indirectly with actual income; it is primarily through insecurity, hopelessness about the future, risk of violence, poor physical health, and limited opportunities that put the poor at high risk for mental health problems [47,48,52].

Food insecurity in high-income countries is associated with increased family stress, violence within the home, and child mental health problems [53–57]. According to qualitative interviews with mothers in Burkina Faso, during times of famine young children display increased symptoms of distress, such as crying [58]. Parenting also changes during the dry season, with female caregivers reporting increased household anxiety and anger sometimes directed toward children [58].

#### Child labor

Extreme poverty also contributes to child labor and heightens children’s risk of experiencing violence and exploitation. In the context of abject poverty, approximately 1.25 million (or 37.8%) of children aged 5–14 in Burkina Faso work to augment their family’s income [59,60]. In addition, some children are sent away to work in gold mines, cotton fields, or cocoa plantations in the South of Burkina Faso, Ivory Coast, or other neighboring countries, often under hazardous conditions [59,60]. Children who work may also be subject to emotional, sexual, and physical violence, with poorly understood emotional and behavioral consequences [61]. Child laborers in Ethiopia show greater psychiatric morbidity than non-economically active children [62], but to our knowledge no study has examined the association between child labor and child mental health outcomes in Francophone West Africa, where child labor is among the world's most prevalent [63].

In addition, gender disadvantage and poverty puts girls from poor families at a higher risk of early marriage [64]. Girls are often faced with early and forced marriage as families stand to gain economically from the reduced financial burden as well as the payment of a bride price [51]. Some West African nations have the highest rate of early marriage for girls under 15 years, and the highest rate of girl children aged 5 to14 years working as maids or domestic workers [50]. Anecdotal evidence suggest that boys in so-called Quranic schools are often made to do unpaid and/or hazardous work including begging in the street and are subject to physical abuse. Children are often sent religious schools because families cannot afford tuition in conventional schools [51].

Thus, using a sample of ultra-poor children living in the Nord Region of Burkina Faso, the paper aims 1) to identify patterns of exposure to four categories of early childhood adversities (violence at home and in the community, child labor and violence at work, and socio-economic deprivation) and 2) to test associations between these groups and child mental health outcomes (depression, trauma symptoms, and self-esteem). Based on previous research, we hypothesized that children who experienced adversities would show greater psychiatric morbidity than those who had not.

## Materials and Methods

### Data Source

This paper uses baseline data from a three-arm cluster randomized control trial evaluating an economic empowerment intervention, alone and in combination with a child rights sensitization component, conducted in the Nord Region of Burkina Faso and designed to prevent family separation and children’s exposure to violence and exploitation (ClinicalTrial.gov ID: NCT02415933). The University of Chicago Institutional Review Board (IRB13-1481) and the Ethics Committee for Research in Health (ECRH), Ministry of Scientific Research and Innovation, Burkina Faso, gave ethical approval for all study and consent procedures.

### Study Participants

The sample consists of 360 mothers (all currently married) and 360 of their children recruited from the poorest villages in the Nord Region of Burkina Faso. The Nord Region is located near the Sahel Desert and is characterized by extreme poverty and ongoing cyclical food crises due to the arid climate. Participants were selected in three stages. First, twelve impoverished comparable villages were selected in the Nord Region based on socio-economic indicators, including poorest yield for crops and limited access to schools and clean water; limited presence of non-governmental organizations implementing development projects; geography (e.g., distance from urban center); similar population size; and ethnic homogeneity. Second, within the selected twelve villages, households living in ultra-poverty (the poorest of the poor households) were identified using a Participatory Wealth Ranking (PWR) exercise, developed by Trickle Up, a local implementing partner, in which community members themselves determined the main characteristics of poverty level, followed by household-level verification of poverty status (e.g., ownership of livestock, productive equipment, land, housing construction, and number of dependents).

Finally, within each household one female caregiver was recruited if she cared for a child between the ages of 10–15. Households with 10–15 year olds were selected because this is the age range deemed most vulnerable to poor child protective outcomes in this area (e.g., separation from family due to work, early marriage, and dropping out of school). Given the local culture, consent for participation was taken first from male heads of households and then short written consent forms were obtained from the female caregiver and her eligible child. Children completed an informed assent form, separately from their parents, to avoid possible coercion. If a female caregiver refused to participate in the study, the household was not enrolled in the study. If her eligible child refused to participate in the study, the child was not enrolled in the study. For illiterate participants, an informed consent/assent procedure involved a witness who could read and write. Depending on the household, the witness could be an educated member of the household, an educated relative living outside of the household, or an educated community member. To avoid the possible coercion, the witness was someone trusted and nominated by the potential participant. Due to low literacy level among the potential participants, a signature in the form of finger paints was accepted.

In the case of polygamous households, the male head-of-household was requested to nominate the poorest wife. If a different wife had been identified through the PWR exercise, then the poorest wife was requested to participate instead. If the participating caregiver had more than one eligible child, we randomly selected the child based on whose birthday was closest to the interview date. In total, 360 ultra-poor households (30 per village) were identified. The number of participants per village was based on the intervention methodology. Women’s savings groups are designed to have about 25–30 members per group. Eligible and consenting female caregivers and children (360 women and 360 children) were enrolled in the study.

### Data Collection

**Fig 1:**
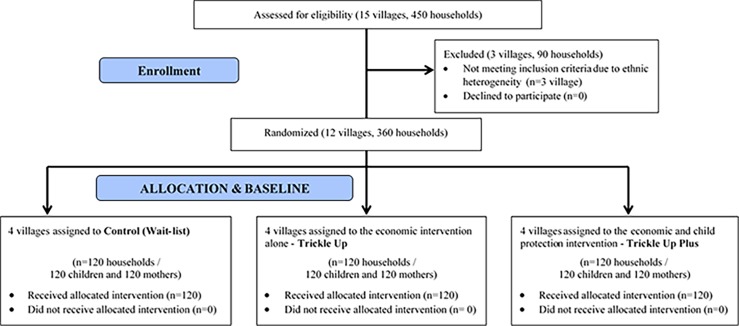
The CONSORT study flow chart.

Baseline data were collected in October 2014. As shown in the CONSORT Flow Chart (Fig 1), 450 households were screened for participation and 360 households (30 households per village) met the eligibility criteria. No participants refused the participation in the study. Three villages (with 90 households) did not meet selection criteria due to ethnic heterogeneity and were not included in the study.

Interviewer-administered surveys were conducted both with mothers and one of their eligible children. Each interview lasted 30–40 minutes for children and about 60 minutes with mothers and was conducted separately at or near the participant’s home. All standardized mental health measures were available in French. When a French version of other instruments were not available, questionnaires were translated from English into French and the local language, Mòoré. A team of graduate sociology students from the University of Ouagadougou, trained by the principal investigators, obtained informed consent and administered the surveys. All data collectors were bilingual in French and Mòoré. The evaluation tools and research protocol were reviewed and modified for cultural equivalence jointly with the Community Collaborative Board (CCB), which included members from the Ministry of Social Protection, the University of Ouagadougou, and national and regional child protection experts.

### Measures

#### Child Mental Health Outcomes

**Depression:** We assessed the severity of depressive symptoms among children with the 20-item Center for Epidemiological Studies Depression Scale for Children/CES-DC [65]. Children reported how they had felt or acted over the preceding week on a 4-point scale from 0 (not at all) to 3 (a lot) for their level of depressiveness. Items for which high scores indicated happiness were reverse coded so that a higher composite score indicated high depressive symptoms. As in a similar study conducted in the African context, participants scoring at or above the clinical cutoff score of 30 were classified as screening positive for depression [66]. The CES-DC demonstrated good internal consistency in this sample (Cronbach α = 0.859).

**Trauma symptoms:** The Children’s Revised Impact of Events Scale/CRIES-8 [67] was used to assess children’s post-traumatic stress reactions over the preceding seven days. The CRIES-8 is a widely used screening tool for post-traumatic stress symptoms that has been translated into 21 languages, including French, and has been tested in many developing countries following natural and manmade disasters [68,69]. Although there are other validated tools for measuring post-traumatic stress disorder (PTSD) among children (e.g., Child PTSD Symptom Scale [70] or UCLA PTSD Index for DSM IV-Adolescent version; [71]), we selected CRIES-8 because the tool is brief and easily understood by participants with low literacy [72]. The CRIES-8 has two subscales, each with four questions: Intrusion (measuring children’s experiences of intrusive thoughts related to traumatic events, e.g., *Do you think about it even when you don’t mean to?*) and Avoidance (measuring avoidance of circumstances and feelings that would remind them of traumatic events, e.g., *Do you try not talk about it?*). Responses to the eight items were rated on a 4-point scale of 0 (not at all), 1 (rarely), 3 (sometimes), and 5 (often). Intrusion and avoidance subscale scores were calculated by adding the item scores within each subscale. The maximum possible total composite score (Intrusion + Avoidance) is 40 (Cronbach α = 0.939), and a clinical cut-off score of 17 was used to screen for symptoms of PTSD [69].

**Self-esteem:** The Rosenberg Self-Esteem Scale/RSES [73] is a 10-item scale measuring children’s global feelings of self-worth or self-regard. Children rated a series of statements on a 4-point Likert-type scale of 0 (strongly disagree) to 3 (strongly agree). Negative items were reverse-coded and higher score indicates high self-esteem. The composite score ranges from 0–30, where higher scores indicate higher levels of global self-esteem (Cronbach α = 0.759). The RSES is available in French and has been validated in multiple country contexts, including the Sub-Saharan African countries of Botswana, the Democratic Republic of the Congo, Ethiopia, Tanzania, and Zimbabwe [74].

#### Adverse Childhood Experiences

**Children’s exposure to violence (reported by children):** The Child Abuse Screening Tool Children’s Version (ICAST-C), developed by the International Society for the Prevention of Child Abuse and Neglect (ISPCAN) [75], and items adapted from the Adverse Childhood Experiences (ACE) Study [27] were used to identify children’s lifetime and current exposure to physical and emotional abuse. A binary measure of physical abuse was created based on children’s exposure to any of six acts (Has any adult… *pushed*, *kicked*, *grabbed*, *shoved*, *slapped you*, *or thrown something at you?* *hit*, *beat*, *or spanked you with a belt*, *paddle*, *a stick or other object?* *suffocated/choked you*, *tried to drown or burn you?* *pulled your hair*, *pinched you roughly*, *or roughly twisted your ear?* *threatened you with a knife*, *gun or any other weapon?* *hit you so hard that you had marks or were injured?*). Likewise, a binary measure of emotional abuse was constructed from four items (*shouted*, *yelled*, *or screamed at you very loud and aggressively?* *called you names*, *said mean things or cursed you?* *made you feel humiliated or very bad in front of other people?* *threatened you in a way that made you afraid you might be physically hurt?*). Children who reported current violence (within the past year) also indicated whether a family member, someone at work, or someone outside the home (in the brush, fields, or village) committed the abuse. The ICAST-C was created for international use and is validated in multiple country contexts [75]. The item “*spanked on the bottom with bare hand*” was removed per CCB’s suggestion as irrelevant to the Burkina Faso context. Two items from the ACE instrument were added to measure violence resulting in injuries and threats of physical violence.

**Children’s exposure to harsh discipline (reported by caregivers):** Mothers’ reports of children’s exposure to harsh discipline practices were assessed using an adapted version of the UNICEF Multiple Indicator Cluster Survey (MICS4), Household Questionnaire. The Child Discipline scale (containing 11 items to measure harsh discipline practices) asked mothers whether they or a member of the household had engaged in violent or harsh discipline practices in the past year (e.g., *Hitting or slapping the child with bare hands or an object; shouting*, *yelling*, *or screaming at the child; calling the child dumb*, *lazy*, *or another name like that; shaming the child by having him or her stand on their knees; depriving the child from food; and beating the child up*, *or hitting the child over and over as hard as one could*). Answers in the affirmative were coded as 1 and negative answers were coded as 0. Two items pertaining to non-violent discipline practices (explaining to a child why a behavior was wrong; taking away privileges) were not included in the analysis. The MICS has been used in multiple countries, including Burkina Faso [76], to evaluate the national status of child well-being and prevalence of child abuse [77,78].

**Children’s exposure to domestic violence:** Items measuring domestic violence in the family were adapted from the Demographic and Health Survey (DHS), previously tested in the Burkina Faso context [79]. Binary measure of mother’s report of physical domestic violence in the past year *included if a woman’s husband had shaken*, *pushed her or threw an object at her*, *slapped her or twisted her arm*, *hit her with his fist or with an object that could have hurt her*, *kicked her or pulled her*, *tried to strangle her or to burn her*, *threatened her with a knife*, *firearm or any other type of weapon*, *attacked her with a knife*, *firearm or another type of weapon)*. Each item was coded as “1” if woman had experienced violent act ‘sometimes’ or ‘often’ in the past year, and “0” if she had not experienced it within the past year. If a mother answered “sometimes” or “often” for any physical violence item within the past year, she was considered to have suffered from current physical domestic violence.

**Child labor and abuse at work:** Questions adapted from National Child Labor Surveys developed for use in low-income countries by the International Labour Organization’s Statistical Information and Monitoring Programme on Child Labour (ILO-SIMPOC) assessed the children’s exposure to child labor [80]. For children aged 5–11 years, UNICEF defines “child labor” as spending more than 28 hours a week on household chores (e.g. cooking, washing clothes, cleaning dishes, collecting water or wood) or being involved in any economic activity for at least one hour a week; for children ages 12–14, those involved in economic activity for more than 14 hours a week are considered child laborers [81]. Children reported in which economic activities they had participated during the past 12 months (such as helping with small business, fishing, hunting, construction work, or agriculture) and working conditions (working hours; unpaid work vs. work for pay or in-kind). Given a large number of child labor activities, we performed the Principle Component Analysis (PCA) for data reduction purposes. Based on the PCA eigenvalues and component loadings, economic activities were grouped into two main categories: working for your own family and working for somebody else. Engaging in animal husbandry loaded on a separate factor and was included as a single item. Fetching water or collecting wood for household use did not load on the main economic factors and were included as household chores as suggested by the ILO classification. Questions measuring hazardous work (defined by the ILO as conditions dangerous for children’s safety or health) included whether children *carried heavy loads*, *operated heavy machinery or used dangerous tools that could lead to a serious injury such as tractor*, *knives or machetes*, *worked underground*, *with toxins*, *chemicals or explosives*, *during inclement weather*, *with fire*, *or under other dangerous conditions*.

#### Socio-Economic Hardship and Deprivation

**Food insecurity:** A measure of food insecurity was adapted from the Household Hunger Scale/HHS [82], which has been used in the Sub-Saharan African countries of Ethiopia, Mozambique, Kenya, South Africa, Zimbabwe, and Malawi. The 4-item instrument asked children how frequently they had experienced food deprivation over the past month: never, rarely (1–2 times), sometimes (3–10 times) or often (more than 10 times). The items included: *ate a smaller meal than you felt you needed because there was not enough food; ate fewer meals in a day because there was not enough food; went to sleep at night hungry because there was not enough food;* and *went for a whole day and night without eating anything because there was not enough food*. Responses were summed such that higher scores represent greater food insecurity. The severity of hunger (with a minimum possible score of 0 and a maximum possible score of 12) was recoded into three categories: little to no household hunger (0–5), moderate hunger (6–7), and severe hunger (8–12) with cutoff points set at the mean and 75^th^ percentile.

**Household poverty:** Household poverty was measured using an adapted household wealth index from the Demographic Health Survey/DHS [83] and the Niger 2011 National Survey on Household Living Conditions and Agriculture [84]. The DHS household wealth index has been used in over 90 developing countries, particularly in countries with irregular income data, and the ECVM/A was developed in collaboration with the World Bank for use in Francophone West Africa. Mothers were queried about ownership of household assets including durable goods and transportation (e.g., electricity, radio, set of plates, mobile phone, and bicycle), type of housing materials (natural floor made of soil or sand vs. clay or cement; natural roof made of thatch or banco/wood vs. metal or other more developed materials) as well as ownership of livestock and land for agricultural use. All 16 binary asset items were summed up and grouped into three categories with cutoff points set at the 25^th^ and 75^th^ percentiles. Those scoring below 25^th^ percentile were coded as extreme poor.

*School enrollment* included four categories (classic school, *Madrassa*—combining formal schooling with the study of the Koran, *Quranic school* focusing on religious studies, and no schooling as a measure of deprivation).

#### Socio-demographic covariates

Children’s socio-demographics included gender and age (pre-adolescents ages 10–12 vs. adolescents ages 13–15). Caregiver and household characteristics were reported by mothers and included the mother’s age, ethnicity, education, type of family structure (monogamous or polygamous), and household size. Because families in Burkina Faso have a complex structure, we recorded household size using two measures. The first is the number of people living in a *concession*, a larger household that includes co-wives and extended family members (e.g., in-laws) who reside together on one compound, often in adjacent huts. The second is the number of people in a *ménage*, which includes only persons in the smaller household—the index woman, her husband, and their children—and excludes co-wives, family patriarchs, and other extended family members.

### Data Analysis

First, we compared abused and non-abused children and found no statistically significant differences on key socio-demographic indicators (data not shown). We then conducted a Latent Class Analysis (LCA) to identify patterns of adverse childhood experiences within four domains: exposure to violence in the family, child labor and abuse at work, violence in the community, and socio-economic deprivation. The LCA was performed in Mplus, Version 7.2 [85]. Models were compared based on the Bayesian Information Criterion (BIC) and entropy [85]. Better fitting models have lower BIC values and higher entropy values, which indicates a greater precision in assigning latent class membership. Finally, we used mixed effects (multilevel) regression models to test associations between child mental health outcomes (depression, trauma symptoms, and self-esteem) and latent classes. Regression analysis was conducted in Stata 14.0 [86]. We ran random intercept models adjusting for clustering within villages and accounting for possible within-cluster correlation [87]. The regression models controlled for socio-demographic characteristics—child's age and gender, and family structure. Religion and ethnicity were excluded from the final models due to lack of variations within the sample; participants were overwhelmingly Muslim (98.06%) and of Mossi ethnicity (98.61%). By way of postestimation, the intraclass correlations by village were small (< .1), indicating small between-village variations. All mental health measures have been cross-culturally validated among French-speaking population and show evidence of construct validity. However, these instruments have not been validated in Mòoré language. However, in the current sample all mental health measures correlated in the expected direction (positive associations between scales measuring symptoms of depression and trauma symptoms, r = 0.44 and negative correlations with the self-esteem scale, r = -0.25 and r = -0.14, respectively). In addition to using the cut-off norms from the original child mental health scales, the raw scores were also converted to T-scores. The T-scores over 65 (1.5 standard deviations above the mean) were used to identify the clinical range thresholds within the current sample. The clinical ranges are usually sensitive to the way cut-off thresholds were derived and, therefore, presented in the paper only for descriptive purposes. In regression models, we used continuous scores to test associations between mental health symptomatology and childhood experiences.

## Results

### Socio-demographic Characteristics of the Sample

Children, on average, were 12.6 years old (*SD* = 1.5) and more than half (54%) were boys. About 40% of mothers were in polygamous marriages and only three percent of mothers were able to read and write (Table 1). Children lived in households with up to 27 family members. At baseline, 17% were attending religious schools (Quranic schools or Madrassas). About 5% of girls (n = 8) reported being promised in marriage and their average age was 14.5 years old (SD = 0.76). The date of marriage was set for one girl, in one year.

**Table 1:** Socio-demographic characteristics of the study sample (*N* = 360).

Variable	*Frequency (%)*					
			Gender		Age	
CHILDREN’S SOCIO-DEMOGRAPHIC CHARACTERISTICS	Total		Boys (n = 194)	Girls (n = 166)	Preadolescents (10–12 years) (n = 169)	Early adolescents (13–15 years) (n = 191)
**Promised to marriage** (only among females)	-		n/a	8 (4.82)	0	8 (4.19)
**Age of those promised to marriage** (only among females), *mean*	-		n/a	14.5 (0.76)		
**Literacy** (able to read and write)	194 (53.89)		104 (53.61)	90 (54.22)	88 (52.07)	106 (55.50)
**Education**						
Conventional (classical) school	221 (61.39)		117 (60.31)	104 (62.65)	113 (66.86)	108 (56.54)
Madrassa (formal school which includes the study of the Koran)	50 (13.89)		28 (14.43)	22 (13.25)	22 (13.02)	28 (14.66)
Quranic school (religious school)	12 (3.33)		8 (4.12)	6 (3.61)	7 (4.14)	7 (3.66)
No schooling	84 (23.33)		45 (23.20)	39 (23.49)	31 (18.34)	53 (27.75)
**MOTHER’S SOCIO-DEMOGRAPHIC CHARACTERISTICS**						
**Mother’s age in years**, *mean*	37.13 (SD = 9.60)		37.97 (SD = 9.60)	36.32 (SD = 9.39)	36.38 (SD = 10.01)	37.95 (SD = 9.04)
**Mother’s literary level** (being able to read and write)	10 (2.78)		6 (3.09)	4 (2.41)	7 (4.14)	3 (1.57)
**Marital status**						
Monogamous	218 (60.65)		111 (57.22)	83 (42.78)	105 (62.13)	113 (59.16)
Polygamous	142 (39.44)		83 (42.78)	59 (35.54)	64 (37.87)	78 (40.84)
**Number of other wives in polygamous marriages (*n* = 142)**, *mean*		2 (SD = 0.39)	2.08 (SD = 0.28)	2.19 (SD = 0.51)	2.13 (SD = 0.45)	2.13 (SD = 0.34)
**Age of husband in years**, *mean*	48.73 (SD = 11.48)		50.02 (SD = 11.32)	47.15 (SD = 11.52)	47.63 (SD = 11.85)	49.73 (SD = 11.08)
**Religion**						
Christian	7 (1.94)		3 (1.55)	4 (2.41)	2 (1.18)	5 (2.62)
Muslim	353 (98.06)		191 (98.45)	162 (97.59)	167 (98.82)	186 (97.38)
**Ethnicity**						
Mossi	355 (98.61)		191 (98.45)	164 (98.80)	167 (98.82)	188 (98.43)
Peuhl	4 (1.11)		2 (1.03)	2 (1.20)	1 (0.59)	3 (1.57)
Other (Gourmantche)	1 (0.28)		1 (0.52)	0	1 (0.59)	0
**HOUSEHOLD COMPOSITION**						
**Number of people in a *concession*** (larger household with co-wives and their dependents), *mean*	10.31 (SD = 3.96)		10.39 (SD = 3.98)	10.21 (SD = 3.95)	10.17 (SD = 3.85)	10.43 (SD = 4.06)
**Number of members in a *ménage*** (smaller household consisting of the woman, her spouse, and their dependents and excluding co-wives and their children), *mean*	7.20 (SD = 1.91)		7.31 (SD = 2.09)	7.07 (SD = 1.67)	7.20 (SD = 2.15)	7.20 (SD = 1.67)
**Number of children in the *ménage* under the age of 16**, *mean*	5.72 (SD = 2.49)		5.68 (SD = 2.45	5.76 (SD = 2.54)	5.56 (SD = 2.42)	5.85 (SD = 2.55)

### Child Mental Health

Table 2 shows the mean scores and the percentage of potential cases scoring above the clinical cutoff scores by age and gender. Children’s mean score on the CES-DC, a measure of depressive symptoms, was 20.23 (SD = 8.73), and 14.72% of children exceeded the clinical cutoff (≥ 30), indicating possible cases of depression. Children’s mean score on the CRIES-8 scale, measuring trauma symptoms, was 6.39 (SD = 9.74), and 17.8% of children scored above the clinical cut-off (≥17), indicating possible PTSD. Children’s mean score on the Rosenberg Self-Esteem Scale was 18.81 (SD = 2.77), and 6.39% scored below 15, indicating low self-esteem. There were no significant gender differences in mental health outcomes. Older children (ages 13–15) reported significantly higher symptoms of trauma compared to children ages 10–12 (t = –2.05, p < 0.05). The percentage of children who exceeded the clinical threshold for depression and trauma screening instruments were lower when clinical cutoff scores were derived from T-scores.

**Table 2:** Mental health outcomes among 10–15 year old children from ultra-poor families in Burkina Faso (*N* = 360).

Variables	TOTAL	Boys	Girls	Preadolescents	Early Adolescents
Mental health outcomes	Mean (SD) or Frequency (%)				
CES-DC Depression Score (range 0–60), *mean*	20.23 (SD = 8.73)	20.28 (SD = 8.51)	20.16 (SD = 9.00)	19.96 (SD = 8.89)	20.46 (SD = 8.60)
CES-DC raw score > 30, (%)	53 (14.72%)	27 (13.92%)	26 (15.66%)	25 (14.79%)	28 (14.66%)
Clinical range (T-score >65)	33 (9.17%)	17 (8.76%)	16 (9.64%)	19 (11.24%)	14 (7.33%)
Child Impact of Events Scale / CRIES-8 (range 0–40), *mean*	6.39 (9.74)	6.80 (10.22)	5.91 (9.16)	5.28 (8.87)	7.38 (10.38)
Intrusion sub-scale, *mean*	3.07 (5.00)	3.26 (5.10)	2.86 (4.88)	2.55 (4.71)	3.53 (5.21)
Avoidance sub-scale, *mean*	3.32 (5.27)	3.55 (5.53)	3.05 (4.96)	2.73 (4.76)	3.84 (5.65)
CRIES-8 raw score ≥ 17, (%)	64 (17.78%)	36 (18.56%)	28 (16.87%)	24 (14.20%)	40 (20.94%)
Clinical range (T-score >65),	40 (11.11%)	25 (12.89%)	15 (9.04%)	12 (7.10%)	28 (14.66)
Rosenberg Self-Esteem Scale (range 0–30), *mean*	18.81 (SD = 2.77)	18.91 (SD = 2.72)	18.69 (SD = 2.84)	18.69 (SD = 2.65)	18.92 (SD = 2.89)
Low self-esteem, below 15 score (%)	23 (6.39%)	11 (5.67%)	12 (7.23%)	12 (7.10%)	11 (5.76%)
Clinical range (T-score < 35)	23 (6.39%)	11 (5.67%)	12 (7.23%)	12 (7.10%)	11 (5.76%)

*Notes*: CES-DC—Center for Epidemiological Studies Depression Scale for Children; CRIES-8—Children’s Revised Impact of Events Scale; Rosenberg Self-Esteem Scale.

### Adverse Childhood Experiences

Over half of children (56%) reported ever experiencing physical violence and 54% of all children had reported physically violence in the past year (Table 3). The majority of mothers (82%) reported employing at least one form of physical punishment (e.g., *hitting or slapping the child with bare hand or with an object*) and 92% used verbally or emotionally abusive discipline practices (e.g., *yelling or screaming at the child; depriving food*).

**Table 3:** Childhood adversities experienced by 10–15 year old children from ultra-poor families in Burkina Faso (*N* = 360).

Child labor and abuse at work	Frequency (n)	Percentage (%)
Any physical abuse	203	(56.39)
Any emotional abuse	250	(69.44)
Any physical or emotional abuse	270	(75.00)
Only emotional abuse (without physical)	67	(18.61)
**Current abuse in the family**		
Physical abuse by family member (in the past year)	158	(43.89)
Emotional abuse by family member (in the past year)	208	(57.50)
Harsh discipline practices, *reported by mother*		
Shook child	193	(53.61)
Shouted, yelled at or screamed at child	313	(87.19)
Hit or slapped child with bare hand	243	(67.69)
Hit child with hard object	100	(27.78)
Called child names	240	(66.67)
Hit or slapped child on the face, head or ears	193	(53.61)
Hit or slapped child on the hand, arm, or leg	140	(38.89)
Beat child up	37	(10.28)
Shamed child by having him/her stand on their knees	63	(17.50)
Deprived child from food	122	(33.89)
Children exposed to domestic violence (mother reported physical abuse by spouse in the last year), *reported by mother*	39	(11.20)
**Child labor and abuse at work**		
Worked for family	124	(34.44)
Worked for other household	64	(17.78)
Type of economic activities		
Worked for family business without pay	35	(9.72)
Construction work or repaired household equipment	52	(14.44)
Fishing or hunting, for sale or household use	93	(25.83)
Helps with small business	24	(6.67)
Work with animals for the household	51	(14.17)
Domestic work for someone outside the household	17	(4.72)
Any work (excluding domestic work) for pay or in-kind	34	(9.44)
Non-economic activities		
Work related to field work or food gardening	309	(85.83)
Fetch water or collect wood for household use	300	(83.33)
Working conditions (n = 349)		
Paid for work	60	(17.19)
Number of hours worked per week, *mean*	20.71	(SD = 18.98)
Hours worked in the last week		
none	129	(35.83)
< 14 hours	85	(23.61)
14–19	47	(13.06)
20 and more hours	99	(27.50)
Child spends > 28 hours a week on household chores	66	(18.33)
Separated from family (sent away) for work	16	(4.44)
Gold mines	7	(1.94)
Maid / domestic aid	5	(1.39)
Selling goods or other work	4	(1.11)
Exposed to any hazardous labor	170	(47.22)
Carrying heavy loads	89	(25.07)
Operating dangerous tools or equipment (e.g., tractor, knives or machetes)	88	(24.65)
Dust, fumes (including dust from mines)	80	(22.35)
Fire, gas, flames	3	(0.84)
Loud noise or vibration	12	(3.35)
Extreme weather (e.g., extreme heat or humidity)	27	(7.54)
Work underground	18	(5.03)
Work in too dark or confined space	13	(3.63)
Insufficient ventilation	14	(3.91)
Chemicals (e.g., pesticides, fertilizers, glues, mercury, cyanide) or explosives	14	(3.91)
Other	2	(0.56)
Child forced to beg	44	(12.22)
Child forced to steal	1	(0.28)
Exchanged sex for money	1	(0.28)
Physical abuse at work	88	(24.44)
Emotional abuse at work	168	(46.67)
Sexual abuse at work	2	(0.56)
**Violence in the community**			
Physical violence outside of home (in the past year)	72	(20.00)
Emotional violence outside of home (in the past year)	82	(22.78)
**Socio-economic hardship and deprivation**	
Household Assets index, *mean*	6.58	(SD = 2.5)
no or minimal assets (extreme poverty, <25^th^ percentile)	87
average	150
above average	123
Food insecurity index, *mean*	6.1	(SD = 2.5)
minimal / occasional hunger	99	(27.50)
moderate hunger	99	(27.50)
severe hunger (>75^th^ percentile)	162	(45.00)

*Note*. Data refer to frequency (percent), unless specified as mean (standard deviation).

About 44% of children reported being involved economic activities in the past year. Children worked, on average, 20.7 hours per week (SD = 18.98) and about half were exposed to hazardous work (*carrying heavy loads*; *exposed to dust and fumes*, *including dust from mines*). Sixteen children (4.4%) reported being separated from their families in the past year due to work in gold mines, sale of goods, or domestic work in the capital city Ouagadougou. When separated from families, children reported working 40 hours a week on average.

Deprivation was widespread with the overwhelming majority of children reported experiencing chronic hunger. All households in the study fell in the category of ultra-poor. Households owned, on average, six assets (mobile telephone, bicycle, land and livestock among the most common) and none of the households had electricity.

**Fig 2:**
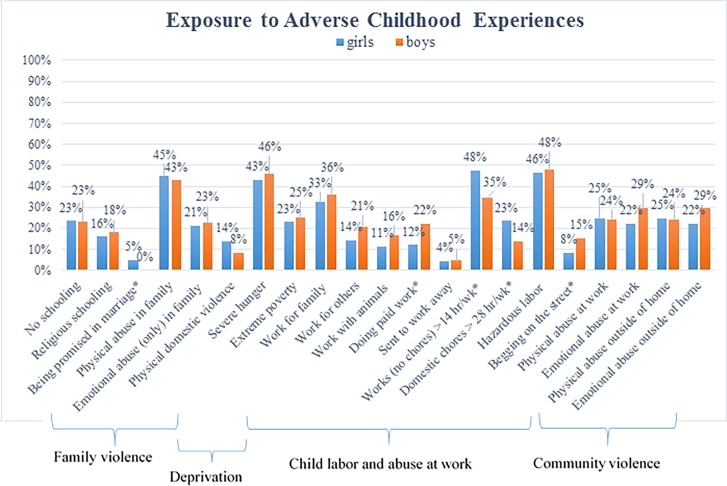
Adverse childhood experiences by gender among 10–15 year old children from ultra-poor families in rural Burkina Faso (*N* = 360). Note: The prevalence numbers statistically different by gender are marked by asterisk (*).

Gender differences in exposure to adverse experiences are presented in Fig 2. Compared to boys, girls were equally involved in economic activities, but fewer girls were paid for their work, and they also worked longer hours and spent more time on household chores; more boys reported begging on the street and had higher reports of physical violence in the community.

### Latent Class Findings

Indicators for the latent classes included exposure to 21 adverse childhood experiences in *the family* (current exposure to physical and emotional abuse in the family, current exposure to domestic violence), *at work* (working for family or for other households, working over 14 hours a week, doing household chores for over 28 hours a week, working away from home, doing paid work, begging and physical and emotional abuse at work), *in the community* (exposure to physical and emotional violence in the community), and exposure to *socio-economic deprivation* (ultra-level poverty, severe hunger, no schooling) as well as exposure to local child protection risks (religious schooling and being promised in marriage). We tested latent class models with a number of other adverse experiences (e.g., exchanging sex for money, stealing, sexual abuse at work), but these indicators had very small values and were removed from the final models.

**Fig 3:**
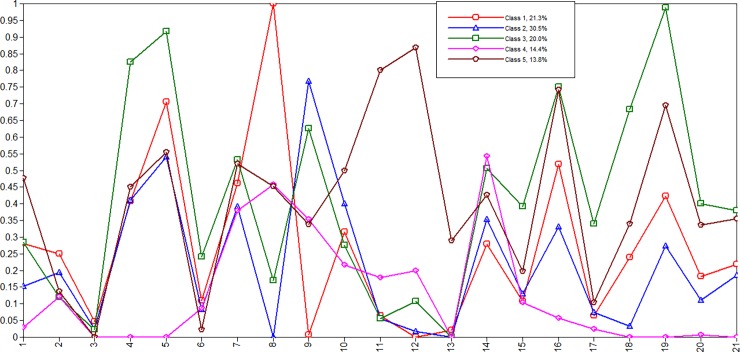
Latent classes of exposure to adverse childhood experiences: Graphical displays of probabilities across each of the five classes. No schooling (1), Religious schooling (2), Being promised in marriage (3), Physical abuse by adult family member (4), Emotional abuse by adult family member (5), Physical domestic violence (6), Severe hunger (7), Extreme poverty (8), Working with animals (9), Working for family (10), Working for other households (11), Doing paid work (12), Sent to work away (13), Working over 14 hours per week (14), Spending more than 28 hours per week on household chores (15) Hazardous labor (16), Begging (17), Physical abuse at work (18), Emotional abuse at work (19), Physical abuse outside of home (20), Emotional abuse outside of home (21).

The LCA models specified and tested 2–6 classes. The best conceptually and statistically fitting model (based on the BIC) was a five-class solution. The entropy value for the five-class model was 0.859, which suggests that there was a good precision in assigning individual cases to the appropriate class. Table 4 includes the response probabilities for each adverse experience by each of the latent classes and these probabilities can be used to describe the five classes. The response probabilities for each experience by class are also graphically presented in Fig 3.

**Table 4:** Latent class analysis among ultra-poor children in Burkina Faso: Probability of latent class membership and item-response probabilities within each of the five classes (N = 360).

Variables	Class 1 Non-working Poor	Class 2 Non-extreme Poor	Class 3 Abused and Exploited	Class 4 Healthy and Non-abused	Class 5 External Laborer
Probability of latent class membership within classes	**(21.32%)**	**(30.53%)**	**(19.96%)**	**(14.42%)**	**(13.78%)**
*Item-response probabilities within each class*					
No schooling	0.281	0.153	0.284	0.030	0.477
Religious schooling	0.250	0.193	0.119	0.121	0.136
Being promised in marriage	0.048	0.025	0.023	0.000	0.000
**Violence in the Family**					
Physical abuse by adult family member	0.407	0.410	0.826	0.000	0.451
Emotional abuse by adult family member	0.706	0.541	0.917	0.000	0.554
Physical domestic violence	0.109	0.084	0.242	0.087	0.023
**Socio-Economic Hardship and Deprivation**					
Severe hunger	0.460	0.391	0.531	0.380	0.520
Extreme poverty	1.000	0.000	0.169	0.457	0.451
**Child labor and abuse at work**					
Working with animals	0.007	0.768	0.625	0.354	0.337
Working for family	0.316	0.400	0.276	0.217	0.499
Working for other households	0.064	0.055	0.055	0.179	0.801
Doing paid work	0.000	0.017	0.108	0.199	0.868
Sent to work away	0.021	0.000	0.000	0.000	0.289
Working over 14 hours per week	0.280	0.354	0.506	0.542	0.426
Spending more than 28 hours per week on household chores	0.108	0.130	0.392	0.105	0.198
Hazardous labor	0.519	0.331	0.750	0.057	0.741
Begging	0.065	0.075	0.340	0.024	0.103
Physical abuse at work	0.239	0.034	0.683	0.000	0.340
Emotional abuse at work	0.423	0.274	0.987	0.000	0.695
**Violence in the community**					
Physical abuse outside of home	0.183	0.111	0.400	0.006	0.336
Emotional abuse outside of home	0.218	0.185	0.379	0.000	0.356
Akaike (AIC)	12861.579				
Bayesian (BIC)	13308.481				
Sample-Size Adjusted BIC	12943.642				

Class 1 (*Non-working Poor*) children in this class show the highest level of poverty, highest probably of obtaining religious schooling, and lowest involvement in child labor, particularly in animal husbandry, and highest probably in being promised in marriage. This class includes over one-fifth (21.3%) of the sample.

Class 2 (*Non-extreme Poor*) children in this class have the lowest probably of experiencing severe hunger and extreme poverty and show the highest involvement in agricultural work (specifically animal husbandry). This is the largest class and represents almost one-third (30.5%) of the sample.

Class 3 (*Abused and Exploited*) children in this class have the highest probability of being physically and emotionally abused at home, being exposed to domestic violence and experiencing abuse in the community. They also have the highest probability of experiencing severe hunger, being engaged in hazardous labor, begging, and spending excessive time on household chores, and experiencing physical and emotional abuse at work. This class represents 20% of the sample.

Class 4 (*Healthy Not abused*) children in this class reported no exposure to abuse in the family, at work or in the community. They spend a lot of hours per week working, but also show the lowest probably of being out of school and less likely to be promised in marriage. This class includes 14.4% of children.

Class 5 (*External Laborer*) includes children with the highest probability of not obtaining any education, working for other households, being sent to work away (e.g., gold mines), and being paid. Children from this class also show the lowest exposure to domestic violence. This class includes 13.8% of the study children.

### Associations between Adverse Childhood Experiences and Child Mental Health

*Non-extreme Poor* was the largest class and was used as the reference group. As shown in Table 5, the regression analysis showed that children from two latent classes—*Abused and Exploited* and *External Laborers*—demonstrated the poorest mental health outcomes (higher depressive and trauma symptoms). Being in the External Laborer class was also associated with significantly lower self-esteem (b = -1.45, SE = 0.48, p<0.01). Children in *Healthy and Non-abused* class demonstrated significantly lower trauma symptoms (b = -3.90, SE = 1.52, p<0.05) and higher self-esteem (b = 0.92, SE = 0.45, p<0.05). Among socio-demographic covariates, only polygamous family structure was associated with greater traumatic symptoms among children (b = 2.44, SE = 1.01, p < 0.05).

**Table 5:** Regression analysis for exposure to childhood adversities and mental health symptoms among children from ultra-poor families in Burkina Faso (*N* = 360).

VARIABLES	Depression^a^	Trauma^b^	Self-esteem^c^
**Latent Classes**	*Estimate (SE)*		
**Class 1: Non-working poor**	2.31	1.87	-0.52
	(1.22)	(1.38)	(0.4)
**Class 2: Non-extreme poor** *(reference group)*	*ref*	*ref*	*ref*
**Class 3: Abused and exploited**	7.18***	4.73***	0.23
	(1.25)	(1.41)	(0.41)
**Class 4: Healthy / non-abused**	-2.13	-3.90*	0.92*
	(1.35)	(1.52)	(0.45)
**Class 5: External laborer**	3.82**	4.25**	-1.45**
	(1.46)	(1.65)	(0.48)
**Covariates**			
**Gender** (girl child)	0.26	-0.35	-0.29
	(0.87)	(0.98)	(0.29)
**Child’s age** (adolescents, 13–15 years of age)	0.13	0.19	0.1
	(0.29)	(0.33)	(0.09)
**Family structure** (polygamous family)	-0.14	2.44*	0.03
	(0.89)	(1.01)	(0.29)
***Constant***	*17*.50***	*3*.*4*	*18*.58***
	(1.64)	(1.83)	(0.52)
**Observations**	360	360	360
**Number of groups**	12	12	12

Notes

^a^Center for Epidemiological Studies Depression Scale for Children (CES-DC)

^b^Children’s Revised Impact of Events Scale (CRIES-8)

^c^Rosenberg Self-Esteem Scale.—Estimates are unstandardized regression coefficients; standard errors of the regression coefficients are in parentheses.

*** p<0.*001*

** p<0.*01*

* p<0.*05*

## Discussion

The study demonstrates that children living with their ultra-poor families in rural Burkina Faso are regularly exposed to a wide range of adverse experiences, with violence from family members, exposure to hazardous labor and food insecurity among the most prevalent. The study also provides evidence that these adverse childhood experiences often co-occur and certain sub-groups of children are exposed to multiple life stressors. The study identified five sub-groups of children that demonstrate distinct profiles of exposure to ACEs: Non-working poor, Non-extreme poor, Abused and Exploited, Healthy Non-Abused and External Laborers. Children in poorest households with limited productive assets are not actively involved in child labor (Non-working Poor), while families with minimal wealth assets (access to land or animals) involve children in economic activities more often (Non-extreme Poor) and represent the largest group in the sample. Violence in the family affected children from a number of classes, but was particularly high among children from the Abused and Exploited class, who were also exposed to violence from multiple sources. Despite living in adverse conditions and working to assist families, one sub-group of children (Healthy and Non-abused class) were not exposed to any violence at home or at work. Children working for external households or away from their own families (External Laborer class) were the smallest group.

Further, the findings showed that about 15% of the children in the study scored above the clinical cut-off for depression, 17.8% for posttraumatic stress disorder (PTSD), and 6.4% for low self-esteem. The estimates derived from the current sample (T-score > 65) showed lower numbers for depression (9%) and trauma (11%). The global prevalence of common mental health conditions among children is about 13% (CI 95% 11.3–15.9)[11] and the estimates for depression from this sample are similar to numbers from other sub-Saharan countries (e.g., 9% in South Africa, 11.9% in Namibia and 14% in Tanzania [88–90]). Nevertheless, the estimates from self-reported measures should be treated with caution, as they tend to overestimate the prevalence of mental conditions compared to clinician-administered diagnostic interviews[11].

Finally, the study showed that accumulation of adverse childhood experiences have strong associations with children’s emotional well-being. Children with exposure to multiple forms of maltreatment at home, at work and in the community (Abused and Exploited class) along with children who are often separated from their families due to work (External Laborer class) showed the poorest mental health outcomes.

However, not all life stressors were associated with negative psychological outcomes. As suggested by the theory of toxic stress [4], children respond to stressful events in three possible ways: positive stress response, tolerable stress response, and toxic stress response. Learning how to cope with adversity is essential for a child’s healthy development and mildly stressful events may help children build resiliency and confidence. For example, while working to assist with a family business may be viewed as a childhood stressor in high-income market-oriented countries, in countries heavily relying on subsistence farming, it may socialize children, particularly adolescents, into productive roles and prepare them for adulthood. However, exposure to prolonged, severe, and frequent stressful events—such as violence, exploitation or severe economic hardship—leads to toxic stress response [4]. The cumulative burden of toxic stress over time (e.g., from chronic child maltreatment), particularly during developmentally sensitive periods, may disrupt a child’s brain functioning and have lifelong changes in that child’s emotional well-being, behavioral self-regulation, and mood control [3,4].

Other studies conducted in sub-Saharan Africa have also demonstrated that exposure to family violence, despite it being highly prevalent and socially accepted, is associated with depression and PTSD among children [34–38,40]. In Tanzania, harsh discipline practices showed a strong association with children’s internalizing problems suggesting that parents underestimate the harmful effects of abusive parenting because children often suffer in silence [43]. Furthermore, studies conducted in Vietnam and Jamaica have demonstrated an association between physical violence and low self-esteem [91,92]. Additionally, children who have been exposed to multiple forms of violence and other stressors such as violence in the community or at school have increased symptoms of PTSD if they are exposed to physical violence within the home [40,93,94]. Some researchers also suggest since one adverse event increases the likelihood of experiencing other adverse events, the clustering of adverse experiences occurs [3,95]. We have observed a similar finding in this study, where children who have experienced violence at home were also more likely to report violence at work and in the community. Despite the putative link between children’s violence exposure and mental health consequences, children in Sub-Saharan Africa are rarely screened for disorders such as depression, anxiety, and post-traumatic stress disorder [8].

Studies of adolescents conducted in other low- and middle-income countries show a strong relationship between exposure to physical abuse and low self-esteem [91,96]. This study provides evidence that emotional abuse, even in the absence of physical violence, is harmful to children’s self-esteem. In West African countries, parents emphasize respect and unquestioning obedience to adults or elders over children’s right to express themselves and voice their opinion [44]. However, immigrants from West Africa admit that although such strict discipline practices prepare children for harsh reality and teach them important moral values such as being responsible and respectful of authority, they also realize that it ‘shuts children down’ [45].

Evidence for the pathways suggests that the association with poverty and mental health is complex. Growing up in a poor household increases the risk of exposure to adversities such as scarcity of food, poor nutrition, violence, and inadequate education, all of which are risk factors for mental disorders [47,97]. However, as evidenced by findings from the current study, living in an ultra-poor household and experiencing severe food insecurity may have an independent association with child’s self-esteem. Ethnographic and quantitative research suggests that food insecurity may not only affect children’s physiological functioning, but may also compromise their cognitive and emotional well-being [98,99]. A qualitative study conducted in Venezuela showed that children were cognitively aware of food insecurity reporting sacrifice in food consumption and recognizing their thinness [55]. They were also preoccupied with feelings of concern and worry for their parents, and expressed anguish, anxiety, and sadness [55]. Ultra-poverty and food insecurity also result in feelings of alienation [e.g., shame) and deprivation (e.g., guilt), and alter household cohesion, leading to family disputes and difficulties keeping children at home [99].

The study has a number of limitations. The study did not utilize a representative sample, interviewed only one child per household who currently resides with their family, and does not include children who are already married or migrated for work. The follow-up data from this study will provide estimates of child marriage and family separation due to work. The study is also limited to currently married females and their children, and its findings may not be generalizable to children growing up without one or both parents or mothers caring for their children without the support of their spouses. Although we used a wide range of adverse childhood experiences to identify the latent classes, these are not the only life stressors and types of maltreatment that children could experience in this context. Latent class analysis is sensitive to indicators that are included and it is possible that including other adverse experiences could show slightly different results. We tested multiple models, although results differed slightly based on the adverse experiences included, the general patterns presented here remained consistent. Finally, the causal nature of the relationships as well as meditating pathways linking childhood psychopathology and adverse childhood experiences should be further examined in longitudinal studies. Positive associations between trauma symptoms and polygamous family structure should not be interpreted causally and should be further explored.

## Conclusions

This study is one of the first studies to explore childhood adverse experiences and its relationships with child psychopathology within the context of Burkina Faso. The findings offer insight into the range of unique adverse experiences faced by children living in ultra-poverty in Francophone West Africa. The study findings suggest that violence in the family is highly prevalent and is strongly associated with negative outcomes for children, but children’s exposure to maltreatment does not exclusively depend on the family poverty level. The study provides initial evidence of the mental health needs of this population and help us better understand how to target the limited mental health promotion and prevention resources. It calls attention to the need for further research to develop integrated economic and mental health interventions for low-income countries that could reduce the burden of socio-economic inequalities on families and prevent children from being exposed to traumatic events detrimental to their development and emotional well-being.

## References

[1] Kessler RC, McLaughlin KA, Green JG, Gruber MJ, Sampson NA, Zaslavsky AM, et al Childhood adversities and adult psychopathology in the WHO World Mental Health Surveys. Br J Psychiatry. 2010;197: 378–385. 10.1192/bjp.bp.110.080499 21037215 PMC2966503

[2] Benjet C. Childhood adversities of populations living in low-income countries: prevalence, characteristics, and mental health consequences. Curr Opin Psychiatry. 2010;23: 356–362. 10.1097/YCO.0b013e32833ad79b 20520546

[3] Anda RF, Felitti VJ, Bremner JD, Walker JD, Whitfield C, Perry BD, et al The enduring effects of abuse and related adverse experiences in childhood. Eur Arch Psychiatry Clin Neurosci. 2006;256: 174–186. 10.1007/s00406-005-0624-4 16311898 PMC3232061

[4] Shonkoff JP, Garner AS, Siegel BS, Dobbins MI, Earls MF, McGuinn L, et al The lifelong effects of early childhood adversity and toxic stress. Pediatrics. 2012;129: e232–e246. 10.1542/peds.2011-2663 22201156

[5] UNICEF. The state of the world’s children UNICEF; 2015.

[6] Izutsu T, Tsutsumi A, Minas H, Thornicroft G, Patel V, Ito A. Mental health and wellbeing in the Sustainable Development Goals. Lancet Psychiatry. 2015;2: 1052–1054. 10.1016/S2215-0366(15)00457-5 26613844

[7] Belfer ML. Child and adolescent mental disorders: the magnitude of the problem across the globe. J Child Psychol Psychiatry. 2008;49: 226–236. 10.1111/j.1469-7610.2007.01855.x 18221350

[8] Cortina MA, Sodha A, Fazel M, Ramchandani PG. Prevalence of child mental health problems in sub-saharan africa: A systematic review. Arch Pediatr Adolesc Med. 2012;166: 276–281. 10.1001/archpediatrics.2011.592 22393184

[9] Kieling C, Baker-Henningham H, Belfer M, Conti G, Ertem I, Omigbodun O, et al Child and adolescent mental health worldwide: evidence for action. The Lancet. 2011;378: 1515–1525. 10.1016/S0140-6736(11)60827-1 22008427

[10] Betancourt TS, Meyers-Ohki SE, Charrow A, Hansen N. Mental Health and Resilience in HIV/AIDS-Affected Children: A Review of the Literature and Recommendations for Future Research. J Child Psychol Psychiatry. 2013;54: 423–444. 10.1111/j.1469-7610.2012.02613.x 22943414 PMC3656822

[11] Polanczyk GV, Salum GA, Sugaya LS, Caye A, Rohde LA. Annual Research Review: A meta-analysis of the worldwide prevalence of mental disorders in children and adolescents. J Child Psychol Psychiatry. 2015;56: 345–365. 10.1111/jcpp.12381 25649325

[12] Meinck F, Cluver LD, Boyes ME, Mhlongo EL. Risk and Protective Factors for Physical and Sexual Abuse of Children and Adolescents in Africa A Review and Implications for Practice. Trauma Violence Abuse. 2015;16: 81–107. 10.1177/1524838014523336 24648489

[13] Nichols J, Embleton L, Mwangi A, Morantz G, Vreeman R, Ayaya S, et al Physical and sexual abuse in orphaned compared to non-orphaned children in sub-Saharan Africa: A systematic review and meta-analysis. Child Abuse Negl. 2014;38: 304–316. 10.1016/j.chiabu.2013.09.012 24210283 PMC3965611

[14] Ssewamala FM, Neilands TB, Waldfogel J, Ismayilova L. The impact of a comprehensive microfinance intervention on depression levels of AIDS-orphaned children in Uganda. J Adolesc Health. 2012;50: 346–352. 10.1016/j.jadohealth.2011.08.008 22443837 PMC3314188

[15] Ssewamala FM, Han C-K, Neilands TB. Asset ownership and health and mental health functioning among AIDS-orphaned adolescents: Findings from a randomized clinical trial in rural Uganda. Soc Sci Med. 2009;69: 191–198. 10.1016/j.socscimed.2009.05.019 19520472 PMC2819297

[16] Ssewamala FM, Nabunya P, Ilic V, Mukasa MN, Ddamulira C. Relationship Between Family Economic Resources, Psychosocial Well-being, and Educational Preferences of AIDS-Orphaned Children in Southern Uganda: Baseline Findings. Glob Soc Welf. 2015;2: 75–86. 10.1007/s40609-015-0027-z 26146601 PMC4486644

[17] Betancourt TS, Rubin-Smith JE, Beardslee WR, Stulac SN, Fayida I, Safren S. Understanding locally, culturally, and contextually relevant mental health problems among Rwandan children and adolescents affected by HIV/AIDS. AIDS Care. 2011;23: 401–412. 10.1080/09540121.2010.516333 21271393 PMC3057405

[18] Betancourt TS, Meyers-Ohki S, Stulac SN, Barrera E, Mushashi C, Beardslee WR. Nothing can defeat combined hands (Abashize hamwe ntakibananira): Protective Processes and Resilience in Rwandan Children and Families Affected by HIV/AIDS. Soc Sci Med 1982. 2011;73: 693–701. 10.1016/j.socscimed.2011.06.053 21840634 PMC3162991

[19] Earls F, Raviola GJ, Carlson M. Promoting child and adolescent mental health in the context of the HIV/AIDS pandemic with a focus on sub-Saharan Africa. J Child Psychol Psychiatry. 2008;49: 295–312. 10.1111/j.1469-7610.2007.01864.x 18221344

[20] Nabunya P, Ssewamala FM. The Effects of parental loss on the psychosocial wellbeing of AIDS-orphaned children living in AIDS-impacted communities: Does gender matter? Child Youth Serv Rev. 2014;43: 131–137. 10.1016/j.childyouth.2014.05.011 25067869 PMC4107308

[21] Skovdal M, Daniel M. Resilience through participation and coping-enabling social environments: the case of HIV-affected children in sub-Saharan Africa. Afr J AIDS Res. 2012;11: 153–164. 10.2989/16085906.2012.734975 24482634 PMC3898544

[22] Betancourt TS, Agnew-Blais J, Gilman SE, Williams DR, Ellis BH. Past horrors, present struggles: The role of stigma in the association between war experiences and psychosocial adjustment among former child soldiers in Sierra Leone. Soc Sci Med 1982. 2010;70: 17–26. 10.1016/j.socscimed.2009.09.038 19875215 PMC3756934

[23] Betancourt TS, McBain R, Newnham EA, Brennan RT. Context matters: community characteristics and mental health among war-affected youth in Sierra Leone. J Child Psychol Psychiatry. 2014;55: 217–226. 10.1111/jcpp.12131 24102324 PMC3944104

[24] Betancourt TS, Speelman L, Onyango G, Bolton P. Psychosocial Problems of War-Affected Youth in Northern Uganda: A Qualitative Study. Transcult Psychiatry. 2009;46: 238–256. 10.1177/1363461509105815 19541749 PMC2775515

[25] Meinck F, Cluver LD, Boyes ME, Ndhlovu LD. Risk and Protective Factors for Physical and Emotional Abuse Victimisation amongst Vulnerable Children in South Africa. Child Abuse Rev. 2015;24: 182–197. 10.1002/car.2283

[26] Djeddah C, Facchin P, Ranzato C, Romer C. Child abuse: current problems and key public health challenges. Soc Sci Med. 2000;51: 905–915. 10.1016/S0277-9536(00)00070-8 10972434

[27] Felitti MD FVJ, Anda MD MRF, Nordenberg MD D, Williamson MS PDF, Spitz MS MAM, Edwards BA V, et al Relationship of Childhood Abuse and Household Dysfunction to Many of the Leading Causes of Death in Adults: The Adverse Childhood Experiences (ACE) Study. Am J Prev Med. 1998;14: 245–258. 10.1016/S0749-3797(98)00017-8 9635069

[28] Gershoff ET. Corporal punishment by parents and associated child behaviors and experiences: A meta-analytic and theoretical review. Psychol Bull. 2002;128: 539–579. 10.1037/0033-2909.128.4.539 12081081

[29] Gershoff ET. Spanking and Child Development: We Know Enough Now to Stop Hitting Our Children. Child Dev Perspect. 2013;7: 133–137. 10.1111/cdep.12038 24039629 PMC3768154

[30] Oladeji BD, Makanjuola VA, Gureje O. Family-related adverse childhood experiences as risk factors for psychiatric disorders in Nigeria. Br J Psychiatry. 2010;196: 186–191. 10.1192/bjp.bp.109.063677 20194539 PMC2830054

[31] McLoyd VC, Smith J. Physical Discipline and Behavior Problems in African American, European American, and Hispanic Children: Emotional Support as a Moderator. J Marriage Fam. 2002;64: 40–53. 10.1111/j.1741-3737.2002.00040.x

[32] Ellison CG, Bradshaw M. Religious Beliefs, Sociopolitical Ideology, and Attitudes Toward Corporal Punishment. J Fam Issues. 2009;30: 320–340. 10.1177/0192513X08326331

[33] Lansford J. The Special Problem of Cultural Differences in Effects of Corporal Punishment. Law Contemp Probl. 2010;73: 89–106.

[34] Ani CC, Grantham-McGregor S. Family and personal characteristics of aggressive Nigerian boys: Differences from and similarities with Western findings. J Adolesc Health. 1998;23: 311–317. 10.1016/S1054-139X(98)00031-7 9814393

[35] Chinawa JM, Aronu AE, Chukwu BF, Obu HA. Prevalence and pattern of child abuse and associated factors in four secondary institutions in Enugu, Southeast Nigeria. Eur J Pediatr. 2013;173: 451–456. 10.1007/s00431-013-2191-4 24197668

[36] Fincham DS, Altes LK, Stein DJ, Seedat S. Posttraumatic stress disorder symptoms in adolescents: risk factors versus resilience moderation. Compr Psychiatry. 2009;50: 193–199. 10.1016/j.comppsych.2008.09.001 19374961

[37] Flisher AJ, Dawes A, Kafaar Z, Lund C, Sorsdahl K, Myers B, et al Child and adolescent mental health in South Africa. J Child Adolesc Ment Health. 2012;24: 149–161. 10.2989/17280583.2012.735505 25860182

[38] Hecker T, Hermenau K, Isele D, Elbert T. Corporal punishment and children’s externalizing problems: A cross-sectional study of Tanzanian primary school aged children. Child Abuse Negl. 2014;38: 884–892. 10.1016/j.chiabu.2013.11.007 24360761

[39] Peltzer K. Posttraumatic stress symptoms in a population of rural children in south africa. Psychol Rep. 1999;85: 646–650. 10.2466/pr0.1999.85.2.646 10611795

[40] du Plessis B, Kaminer D, Hardy A, Benjamin A. The contribution of different forms of violence exposure to internalizing and externalizing symptoms among young South African adolescents. Child Abuse Negl. 2015;45: 80–89. 10.1016/j.chiabu.2015.02.021 25804436

[41] Seedat S, Nyamai C, Njenga F, Vythilingum B, Stein DJ. Trauma exposure and post-traumatic stress symptoms in urban African schools. Br J Psychiatry. 2004;184: 169–175. 10.1192/bjp.184.2.169 14754831

[42] Omigbodun O, Bakare K, Yusuf B. Traumatic events and depressive symptoms among youth in Southwest Nigeria: a qualitative analysis. Int J Adolesc Med Health. 2008;20: 243–253. 19097561 10.1515/ijamh.2008.20.3.243

[43] Hecker T, Hermenau K, Salmen C, Teicher M, Elbert T. Harsh discipline relates to internalizing problems and cognitive functioning: findings from a cross-sectional study with school children in Tanzania. BMC Psychiatry. 2016;16: 1. 27129400 10.1186/s12888-016-0828-3 PMC4850652

[44] Rasmussen A, Akinsulure-Smith A, Chu T, Keatley E. “911” among West African immigrants in New York City: A qualitative study of parents’ disciplinary practices and their perceptions of child welfare authorities. Soc Sci Med. 2012;75: 516–525. 10.1016/j.socscimed.2012.03.042 22591826 PMC3367065

[45] Onwujuba C, Marks L, Nesteruk O. Why We Do What We Do: Reflections of Educated Nigerian Immigrants on their Changing Parenting Attitudes and Practices. Fam Sci Rev. 2015;20 Available: http://www.familyscienceassociation.org/sites/default/files/2%20-%20Onwujuba,%20Nigerian%20immigrant%20parenting.pdf

[46] Patel V, Kleinman A. Poverty and common mental disorders in developing countries. Bull World Health Organ. 2003;81: 609–615. 14576893 PMC2572527

[47] Lund C, Breen A, Flisher AJ, Kakuma R, Corrigall J, Joska JA, et al Poverty and common mental disorders in low and middle income countries: a systematic review. Soc Sci Med. 2010;71: 517–528. 10.1016/j.socscimed.2010.04.027 20621748 PMC4991761

[48] Kinyanda E, Woodburn P, Tugumisirize J, Kagugube J, Ndyanabangi S, Patel V. Poverty, life events and the risk for depression in Uganda. Soc Psychiatry Psychiatr Epidemiol. 2011;46: 35–44. 10.1007/s00127-009-0164-8 19916062 PMC3432478

[49] Bank W. World Development Indicators 2015 [Internet]. Washington, DC; 2015. Available: https://openknowledge.worldbank.org/handle/10986/21634

[50] UNICEF. Child in crisis in the Sahel: Burkina Faso, Cameroon, Chad, Gambia, Mali, Mauritania, Niger, Nigeria, Senegal [Internet]. UNICEF; 2012. Available: http://www.unicef.org/health/files/UNICEF_SAHEL_EmrgRprt_11.12.12.pdf

[51] Population Council. Key Data Points on Adolescent Women in Nord and Sahel regions of Burkina Faso. Population Council; 2012.

[52] Patel V. Poverty inequality and mental health in developing countries. 2001; Available: http://www.popline.org/node/183645

[53] Melchior M, Chastang J-F, Falissard B, Galéra C, Tremblay RE, Côté SM, et al Food Insecurity and Children’s Mental Health: A Prospective Birth Cohort Study. PLoS ONE. 2012;7: e52615 10.1371/journal.pone.0052615 23300723 PMC3530436

[54] McLaughlin KA, Green JG, Alegría M, Jane Costello E, Gruber MJ, Sampson NA, et al Food Insecurity and Mental Disorders in a National Sample of U.S. Adolescents. J Am Acad Child Adolesc Psychiatry. 2012;51: 1293–1303. 10.1016/j.jaac.2012.09.009 23200286 PMC3632292

[55] Bernal J, Frongillo EA, Herrera H, Rivera J. Children Live, Feel, and Respond to Experiences of Food Insecurity That Compromise Their Development and Weight Status in Peri-Urban Venezuela. J Nutr. 2012;142: 1343–1349. 10.3945/jn.112.158063 22623397

[56] Alaimo K, Olson CM, Frongillo EA. Food Insufficiency and American School-Aged Children’s Cognitive, Academic, and Psychosocial Development. Pediatrics. 2001;108: 44–53. 11433053

[57] Huang J, Oshima K, Kim Y. Does household food insecurity affect parenting and children’s behaviors? Evidence from the Panel Study of Income Dynamics (PSID). Soc Serv Rev. 2010;84: 381–401. 20873019 10.1086/655821 PMC4071141

[58] Nanama S, Frongillo EA. Altered social cohesion and adverse psychological experiences with chronic food insecurity in the non-market economy and complex households of Burkina Faso. Soc Sci Med. 2012;74: 444–451. 10.1016/j.socscimed.2011.11.009 22197293

[59] United States Department of Labor, 2013 Findings on the Worst Forms of Child Labor, http://www.dol.gov/ilab/reports/child-labor/findings/2013TDA/burkinafaso.pdf.

[60] Understanding Children’s Work (UCW). Analysis of Child Economic Activity and School Attendance Statistics from National Household or Child Labor Surveys, February, 2012.

[61] Catani C, Schauer E, Elbert T, Missmahl I, Bette J-P, Neuner F. War trauma, child labor, and family violence: Life adversities and PTSD in a sample of school children in Kabul. J Trauma Stress. 2009;22: 163–171. 10.1002/jts.20415 19462436

[62] Fekadu D, Alem A, Hägglöf B. The prevalence of mental health problems in Ethiopian child laborers. J Child Psychol Psychiatry. 2006;47: 954–959. 10.1111/j.1469-7610.2006.01617.x 16930390

[63] United States Department of Labor, 2013 Findings on the Worst Forms of Child Labor, http://www.dol.gov/ilab/reports/child-labor/africa.htm.

[64] Otoo-Oyortey N, Pobi S. Early Marriage and Poverty: Exploring links and key policy issues. Gend Dev. 2003;11: 42–51.

[65] Faulstich ME, Carey MP, Ruggiero L, Enyart P, Gresham F. Assessment of depression in childhood and adolescence: an evaluation of the Center for Epidemiological Studies Depression Scale for Children (CES-DC). Am J Psychiatry. 1986;143: 1024–1027. 10.1176/ajp.143.8.1024 3728717

[66] Betancourt T, Scorza P, Meyers-Ohki S, Mushashi C, Kayiteshonga Y, Binagwaho A, et al Validating the Center for Epidemiological Studies Depression Scale for Children in Rwanda. J Am Acad Child Adolesc Psychiatry. 2012;51: 1284–1292. 10.1016/j.jaac.2012.09.003 23200285 PMC5730330

[67] Children and War Foundation. 2005. Children’s Revised Impact of Event Scale (CRIES-8).

[68] Perrin S, Meiser-Stedman R, Smith P. The Children’s Revised Impact of Event Scale (CRIES): Validity as a Screening Instrument for PTSD. Behav Cogn Psychother. 2005;33: 487–498. 10.1017/S1352465805002419

[69] Yule W, Bruggencate ST, Joseph S. Principal components analysis of the impact of events scale in adolescents who survived a shipping disaster. Personal Individ Differ. 1994;16: 685–691. 10.1016/0191-8869(94)90210-0

[70] Foa EB, Johnson KM, Feeny NC, Treadwell KRH. The Child PTSD Symptom Scale: A Preliminary Examination of its Psychometric Properties. J Clin Child Adolesc Psychol. 2001;30: 376–384. 10.1207/S15374424JCCP3003_9 11501254

[71] Pynoos RS, Rodriguez N, Steinberg AS, Stuber M, & Frederick, C (1998). UCLA PTSD Index for DSM-IV.

[72] Deeba F, Rapee RM, Prvan T. Psychometric properties of the Children’s Revised Impact of Events Scale (CRIES) with Bangladeshi children and adolescents. PeerJ. 2014;2 10.7717/peerj.536 25237597 PMC4157240

[73] Rosenberg M. Society and the Adolescent Self-Image. Princeton, N.J.: Princeton University Press; 1965.

[74] Schmitt DP, Allik J. Simultaneous Administration of the Rosenberg Self-Esteem Scale in 53 Nations: Exploring the Universal and Culture-Specific Features of Global Self-Esteem. J Pers Soc Psychol. 2005;89: 623–642. 10.1037/0022-3514.89.4.623 16287423

[75] Zolotor AJ, Runyan DK, Dunne MP, Jain D, Péturs HR, Ramirez C, et al ISPCAN Child Abuse Screening Tool Children’s Version (ICAST-C): Instrument development and multi-national pilot testing. Child Abuse Negl. 2009;33: 833–841. 10.1016/j.chiabu.2009.09.004 19857897

[76] Akmatov MK. Child abuse in 28 developing and transitional countries—results from the Multiple Indicator Cluster Surveys. Int J Epidemiol. 2011;40: 219–227. 10.1093/ije/dyq168 20943933

[77] Bornstein MH, Britto PR, Nonoyama-Tarumi Y, Ota Y, Petrovic O, Putnick DL. Child Development in Developing Countries: Introduction and Methods. Child Dev. 2012;83: 16–31. 10.1111/j.1467-8624.2011.01671.x 22277004 PMC3412563

[78] Cappa C, Khan SM. Understanding caregivers’ attitudes towards physical punishment of children: evidence from 34 low- and middle-income countries. Child Abuse Negl. 2011;35: 1009–1021. 10.1016/j.chiabu.2011.10.003 22152701

[79] Burkina Faso Demographic and Health Survey 2010. [Internet]. Institut National de la Statistique et de la Démographie (INSD), Ministère de l’Économie et des Finances [Burkina Faso] and ICF International.; 2010. Available: http://dhsprogram.com/pubs/pdf/FR256/FR256.pdf

[80] Ray R, Lancaster G. The impact of children’s work on schooling: multicountry evidence based on SIMPOC data Hobart Univ Tasmânia Sch Econ 2004; Available: http://repec.org/esAUSM04/up.15362.1076562558.pdf

[81] UNICEF—Definitions. http://www.unicef.org/infobycountry/stats_popup9.html.

[82] Ballard T, Coates J; Swindale A, Deitchler M. Household Hunger Scale: Indicator Definition and Measurement Guide. Washington, DC: Food and Nutrition Technical Assistance II Project, FHI 360. [Internet].

[83] Rutstein SO, Johnson K. The DHS Wealth Index. DHS Comparative Reports No. 6. Calverton, Maryland: ORC Macro; 2004. 2011.

[84] ECVM/A. Niger—National Survey on Household Living Conditions and Agriculture 2011 [Internet]. Niger: Survey and Census Division, National Institute of Statistics, the World Bank; 2014 Available: http://microdata.worldbank.org/index.php/catalog/2050

[85] Muthén LK, Muthén BO. Mplus User’s Guide: Statistical Analysis with Latent Variables: User’ss Guide. Muthén & Muthén; 2010.

[86] Stata StataCorp LP. Stata Statistical Software: Release 13. Coll Stn TX StataCorp LP 2013;

[87] Gelman A, Hill J. Data Analysis Using Regression and Multilevel/Hierarchical Models [Internet]. New York: Cambridge University Press; 2012 Available: http://lac.essex.ac.uk/local—files/meetings1213/gelman_1.pdf

[88] Cluver L, Gardner F, Operario D. Psychological distress amongst AIDS-orphaned children in urban South Africa. J Child Psychol Psychiatry. 2007;48: 755–763. 10.1111/j.1469-7610.2007.01757.x 17683447

[89] Ruiz-Casares M, Thombs BD, Rousseau C. The association of single and double orphanhood with symptoms of depression among children and adolescents in Namibia. Eur Child Adolesc Psychiatry. 2009;18: 369–376. 10.1007/s00787-009-0739-7 19198922

[90] Hermenau K, Hecker T, Elbert T, Ruf-Leuschner M. Maltreatment and mental health in institutional care—Comparing early and late institutionalized children in Tanzania. Infant Ment Health J. 2014;35: 102–110. 10.1002/imhj.21440 25798516

[91] Nguyen HT, Dunne MP, Le AV. Multiple types of child maltreatment and adolescent mental health in Viet Nam. Bull World Health Organ. 2010;88: 22–30. 10.2471/BLT.08.060061 20428350 PMC2802435

[92] Smith DE, Springer CM, Barrett S. Physical Discipline and Socioemotional Adjustment Among Jamaican Adolescents. J Fam Violence. 2010;26: 51–61. 10.1007/s10896-010-9341-5

[93] Omigbodun O, Bakare K, Yusuf B. Traumatic events and depressive symptoms among youth in Southwest Nigeria: a qualitative analysis. Int J Adolesc Med Health. 2008;20: 243–254. 19097561 10.1515/ijamh.2008.20.3.243

[94] Collings SJ, Penning SL, Valjee SR. Lifetime Poly-Victimization and Posttraumatic Stress Disorder among School-Going Adolescents in Durban, South Africa. j psychiatry 17: 133 10.4172. Psychiatry. 2014;1000133: 2.

[95] Chapman DP, Whitfield CL, Felitti VJ, Dube SR, Edwards VJ, Anda RF. Adverse childhood experiences and the risk of depressive disorders in adulthood. J Affect Disord. 2004;82: 217–225. 10.1016/j.jad.2003.12.013 15488250

[96] Smith DE, Springer CM, Barrett S. Physical Discipline and Socioemotional Adjustment Among Jamaican Adolescents. J Fam Violence. 2010;26: 51–61. 10.1007/s10896-010-9341-5

[97] Lund C, De Silva M, Plagerson S, Cooper S, Chisholm D, Das J, et al Poverty and mental disorders: breaking the cycle in low-income and middle-income countries. The Lancet. 2011;378: 1502–1514. 10.1016/S0140-6736(11)60754-X 22008425

[98] Melchior M, Chastang J-F, Falissard B, Galéra C, Tremblay RE, Côté SM, et al Food Insecurity and Children’s Mental Health: A Prospective Birth Cohort Study. PLoS ONE. 2012;7: e52615 10.1371/journal.pone.0052615 23300723 PMC3530436

[99] Nanama S, Frongillo EA. Altered social cohesion and adverse psychological experiences with chronic food insecurity in the non-market economy and complex households of Burkina Faso. Soc Sci Med. 2012;74: 444–451. 10.1016/j.socscimed.2011.11.009 22197293

